# Endometrial osseous metaplasia: sonographic, radiological and histopathological findings

**DOI:** 10.1590/0100-3984.2015.0032

**Published:** 2016

**Authors:** Luiz Felipe Alves Guerra, Laís Bastos Pessanha, Gabriel Antonio de Oliveira, Adriana Maria Fonseca de Melo, Flavia Silva Braga, Rodrigo Stênio Moll de Souza

**Affiliations:** 1 Universidade Federal do Espírito Santo (UFES), Vitória, ES, Brazil.

*Dear Editor,*

A 31-year-old, female patient with previous history of spontaneous miscarriage with uterine curettage eight years ago, undergoing investigation for secondary infertility, increased menstrual flow and pelvic pain.

Transvaginal ultrasonography (US) (Figures 1A and 1B) showed a hyperechoic endometrial, nonspecific, plate-shaped image with posterior acoustic shadowing, and measuring 2.7 × 2.6 cm. Pelvic radiography (Figure 1C) identified a focus of calcification at endometrial site.

**Figure 1:**
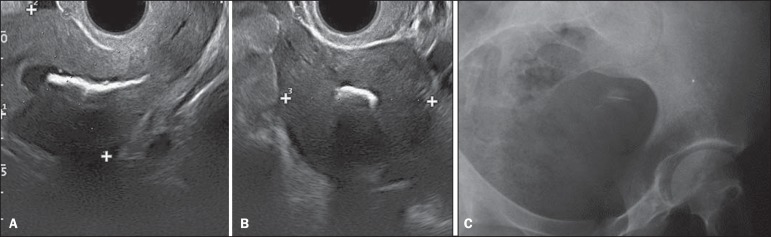
**A,B:** Transvaginal ultrasonography demonstrating hyperechoic image with posterior acoustic shadowing in the endometrium, compatible with calcification. **C:** Hip radiography, oblique view showing an image with calcific density corresponding to the one found at ultrasonography, strengthening the suggested hypothesis.

On the basis of the imaging findings and clinical history, the presumptive diagnosis was endometrial osseous metaplasia, confirmed by histopathological study revealing the presence of a plate of trabecular bone tissue surrounded by fibrous tissue and proliferative endometrium (Figure 2). Cartilage, bone marrow, chronic inflammation and trophoblastic tissue were not present.

**Figure 2:**
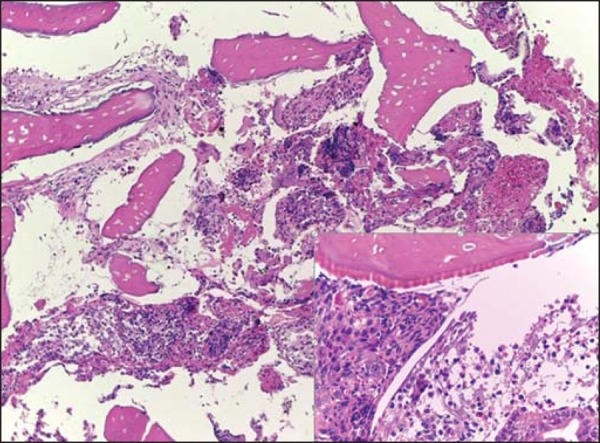
Photomicrography with low and medium magnification showing osseous trabeculae intermingled with endometrial tissue. Observe the endometrial glands at the lower right corner. Hematoxylin-eosin staining.

Endometrial osseous metaplasia corresponds to the presence of bone-like tissue within the uterine cavity. It is a rare entity, affecting only 0.15% of the patients referred to hysteroscopy clinics^(1,2)^. The pathogenesis of such a condition still remains controversial. The two most accepted mechanisms involve either the presence of chronic endometrioses with undifferentiated mesenchymal cells inducing the endometrial stromal cells transformation into osteoblasts, or miscarriage with dystrophic ossification of the residual ovular tissues^(3)^. Such hypotheses are reinforced as one considers that more than 80% of cases occur after pregnancies that evolved to miscarriage, particularly those followed by infection^(4)^. Symptoms include pelvic pain and menstrual flow alterations, but the main consequence of the presence of bone tissue in the uterine cavity is infertility^(5)^. The association between osseous metaplasia and infertility occurs because of the similarity between the action of the bone tissue and the action of an intrauterine contraceptive device (IUCD)^(6,7)^.

The main sonographic finding of endometrial osseous metaplasia is the presence of a strongly echogenic endometrial plate with posterior acoustic shadowing, assuming the presence of an IUD as main differential diagnosis. Other possible diagnoses include: presence of foreign bodies, Asherman's syndrome, calcified submucosal fibrosis and Müllerian tumor^(2,5,6)^. However, the suspicion of endometrial osseous metaplasia should be taken into consideration by the sonographist in cases where strongly echogenic endometrial plates are detected in patients with history of miscarriage and chronic endometriosis.

In the presently reported case, the correlation between transvaginal US and pelvic radiography has allowed for the diagnosis of endometrial calcification. The previous history of miscarriage with curettage has corroborated the hypothesis of calcification corresponding to osseous metaplasia induced by chronic endometritis, which later was confirmed by histopathological analysis of bone fragments collected by means of hysteroscopy.

Transvaginal US is the best imaging method in such cases, since hysterosalpingography and magnetic resonance imaging may miss the findings. In such cases, the investigator must describe the location and the dimensions of the echogenic plate, rule out the presence of an IUCD, and reinforce the history of miscarriage with chronic endometritis, corroborating the hypothesis of metaplastic endometrial ossification. Such informations are important for the hysterocopist who will make the resection of the osseous plate with subsequent histopathological analysis.

The treatment for this condition should be performed by means of hysteroscopic removal of osseous fragments to be submitted to histopathological analysis or, as a second option by uterine curettage^(8)^. In the present case, the first alternative was adopted and the patient had her fertility restored and her menstrual flow reduced.

## References

[1] Parente RCM, Freitas V, Moura RS (2010). Metaplasia óssea endometrial: quadro clínico e
seguimento após tratamento. Rev Bras Ginecol Obstet.

[2] Shalev J, Meizner I, Bar-Hava I (2000). Predictive value of transvaginal sonography performed before
routine diagnostic hysteroscopy for evaluation of
infertility. Fertil Steril.

[3] Shroff CP, Kudterkar NG, Badhwar VR. (1985). Endometrial ossification - report of three cases with literature
review. Indian J Pathol Microbiol.

[4] Pinto AP, Guedes GB, Tuon FFB. (2005). Metaplasia óssea do endométrio: relato de
caso. J Bras Patol Med Lab.

[5] Umashankar T, Patted S, Handigund R. (2010). Endometrial osseous metaplasia: clinicopathological study of a
case and literature review. J Hum Reprod Sci.

[6] Basu M, Mammen C, Owen E (2003). Bony fragments in the uterus: an association with secondary
subfertility. Ultrasound Obstet Gynecol.

[7] Onderoglu LS, Yarali H, Gultekin M (2008). Endometrial osseous metaplasia; an evolving cause of secondary
infertility. Fertil Steril.

[8] Cayuela E, Perez-Medina T, Vilanova J (2009). True osseous metaplasia of the endometrium: the bone is not from
a fetus. Fertil Steril.

